# Imaging biomarkers and clinical factors associated with the rate of progressive inner and outer retinal thinning in patients with diabetic macular edema

**DOI:** 10.1038/s41598-023-30432-2

**Published:** 2023-02-24

**Authors:** Enrico Borrelli, Costanza Barresi, Alessandro Feo, Giorgio Lari, Domenico Grosso, Lea Querques, Riccardo Sacconi, Francesco Bandello, Giuseppe Querques

**Affiliations:** 1 Department of Ophthalmology, IRCCS San Raffaele Scientific Institute, Via Olgettina 60, Milan, Italy; 2 Vita-Salute San Raffaele University, Milan, Italy

**Keywords:** Predictive markers, Diabetes complications

## Abstract

The aim of this study was to assess the relationship of clinical characteristics to the rate of retinal thinning in eyes with diabetic macular edema (DME) treated with anti-vascular endothelial growth factor (VEGF) therapy. We analyzed subjects with a long-term follow-up (≥ 3 years) and evidence of resolved DME after the initiation of anti-VEGF therapy (baseline visit). To measure the long-term rate of retinal thinning during treatment, a second visit (first visit with evidence of resolved DME after 3 years) was also considered. A longitudinal quantitative topographical assessment of the inner and outer retinal thicknesses was provided. Clinical characteristics were associated with the rate of longitudinal retinal thinning. We included 56 eyes (50 patients) in the analysis. A significant longitudinal thinning in the inner and outer retina was detected in all the analyzed regions (p values between 0.027 and < 0.0001). In the multivariable analysis, type of diabetes (type 2 vs. type 1) was associated with increased foveal inner retinal thinning (*p* = 0.019). A higher number of subfoveal neuroretinal detachment during follow-up (*p* = 0.006) was associated with faster rates of foveal outer retinal thinning. Type of diabetes (*p* < 0.0001), higher age (*p* = 0.033) and cystoid macular edema phenotype (*p* = 0.040) were associated with increased parafoveal inner retinal thinning. Gender (*p* = 0.003) and diabetic retinopathy stage (*p* = 0.013) were associated with faster rates of perifoveal inner retinal thinning, while diabetic retinopathy stage (*p* = 0.036) was associated with increased perifoveal outer retinal thinning. In conclusion, clinical factors, including DME phenotypes, were associated with the rates of retinal thinning in patients undergoing anti-VEGF treatment.

## Introduction

Diabetic retinopathy (DR) is a leading cause of visual impairment^1^. Diabetic macular edema (DME) is a common complication as this may occur in approximately 12% of DR patients^2^.

The chronic and progressive nature of DME necessitates timely detection of the disease through analysis of structural and functional changes. Structural optical coherence tomography (OCT) is commonly employed in the management of patients with DME. Furthermore, this imaging modality grants a qualitative and quantitative assessment of structural parameters of the macula with good precision and reproducibility. Although DME is noted for an accumulation of intraretinal fluid with concomitant retinal thickening, this disease may be also characterized by a progressive structural loss of the neuroretina as several previous studies using structural OCT have demonstrated that DME eyes could be featured by a thinning of the inner and outer retinal layers after resolution of intraretinal and subretinal fluid (i.e. resolved DME)^3–7^. More importantly, an injury of the inner and/or outer retina in cases with resolved DME was correlated with a poorer visual outcome in these eyes^4–12^.

Diabetic macular edema can manifest with different morphological patterns on structural OCT. In details, DME eyes may exhibit a diffuse macular thickening with areas of reduced reflectivity mainly located in the outer retina^13,14^. Conversely, DME may be characterized by cystoid spaces (i.e. cystoid macular edema—CME) that appear as round- or oval-shaped areas of lower reflectivity with highly reflective septa isolating these intraretinal cavities accommodating fluid^13–16^. Finally, a subfoveal neuroretinal detachment (SND) may complicate eyes with DME and this finding may be visualized as an hyporeflective space beneath the neuroretina on OCT images^13–16^. More importantly, distinct morphological patterns were characterized by different visual outcomes as presence of cystoid macular edema and/or subfoveal retinal detachment was associated with an overall poorer visual prognosis in eyes with DME^13–16^. Regardless of the OCT pattern, the foveal thickness represents a predictor of visual acuity in eyes affected by DME, as both a smaller and greater foveal thickness is associated with a reduced visual acuity after the resolution of intraretinal fluid^12,17^.

There is a lack of information regarding the relationship between OCT patterns and the rate of retinal thinning in eyes with DME. Therefore, the aim of this study was to provide a longitudinal quantitative analysis of the inner and outer retina in different anatomic phenotypes of patients with early DME followed prospectively for several years.

## Methods

### Subjects

In the present study, patients older than 18 years with a diagnosis of center-involved DME were retrospectively identified at the San Raffaele Scientific institute. Informed consent was gained from all subjects included in the study. The San Raffaele Ethics Committee was informed about this retrospective study that adhered to the 1964 Helsinki declaration and its later amendments. Based on the Italian legislation, this kind of study doesn't require the Ethics Committee approval, as only a notification is required.

Patients underwent a complete ophthalmological assessment, including fundus ophthalmoscopy and structural OCT. The diagnosis of center-involved DME was based on clinical and imaging assessment, as previously reported^18^. Included patients also had history of anti-VEGF injections. The following exclusion criteria were adopted: (i) treatment with macular laser after the initiation of anti-VEGF injections; (ii) previous vitreoretinal procedure in the study eye; (iii) presence of other macular (e.g. vitreoretinal disorders) and/or optic nerve (e.g. glaucoma) pathologic conditions, as they may have an impact on the OCT metrics.

All the included subjects underwent a pro re nata regimen using anti-VEGF intravitreal injections of different drugs (i.e. ranibizumab, aflibercept, or bevacizumab). During the study period, a switch to the intravitreal dexamethasone implant (Ozurdex^®^) was at the treating ophthalmologist’s discretion.

Subjects included in the final cohort of analyzed patients were also needed to have at least 3 yearly clinical assessments over a period of three years following the first confirmation of DME resolution (i.e. reformation of the foveal depression with a foveal thickness inferior to 315 µm)^12,19^ after the initiation of anti-VEGF treatment. The latter visit was considered as the baseline visit in our analysis. In order to provide a longitudinal assessment of the retinal changes, a second visit after 3 years of continued follow-up exams successive to the baseline visit was included in the analysis. The latter visit (i.e. study follow-up visit) was defined as the first visit with OCT confirmation of resolved DME after a follow-up of 3 years. Our analysis was performed at visits characterized by fluid resolution in order to avoid intraretinal fluid from confounding the OCT quantification^20^.

The final study cohort of analyzed patients consisted of 50 subjects (56 eyes) as 6 patients had both eyes included.

The Heidelberg Spectralis HRA + OCT (Heidelberg Engineering, Heidelberg, Germany) device was employed to perform spectral domain OCT. Each volumetric scan was composed of 19 horizontal B-scans, each of which comprised 24 averaged scans, covering an approximately 5.5 × 4.5-mm area centered on the fovea. To be included, scans were also required to be characterized by a minimum signal strength of 25, as advised by the manufacturer^21^.

Visual acuities were tested with Snellen charts and successively transformed to LogMAR (logarithm of the minimal angle of resolution) values.

### OCT assessment

Structural OCT images at baseline and 3-year follow-up visits were first reviewed for eligibility by an expert and certified reader (EB) and a junior grader (CB).

In order to assess longitudinal structural quantitative changes, OCT images at both baseline and follow-up visits were investigated for the following quantitative metrics:

- Inner and outer retinal thicknesses: the manufacturer’s built-in software was employed to measure the thickness of the retina inside the different ETDRS-grid regions (i.e. the central fovea subfield within the inner 1-mm-diameter circle, the inner circle subfield between the inner and middle 3-mm-diameter circles, and the outer circle subfield between the middle and outer 6-mm-diameter circles)^22^. As previously described^22^, this algorithm detects and calculates the thickness of the inner retina (layers between the inner margin of the nerve fiber layer and the inner boundary of the outer plexiform layer). Conversely, the outer retina was measured between the inner boundary of the outer plexiform layer and the inner margin of the retinal pigment epithelium. All B-scans were evaluated by the two readers in order to correct any decentration or segmentation errors before calculating the thickness values. Cases with layers’ disruption causing an inability to detect boundaries to calculate retinal thicknesses were not included in the analysis.

**Figure 1:**
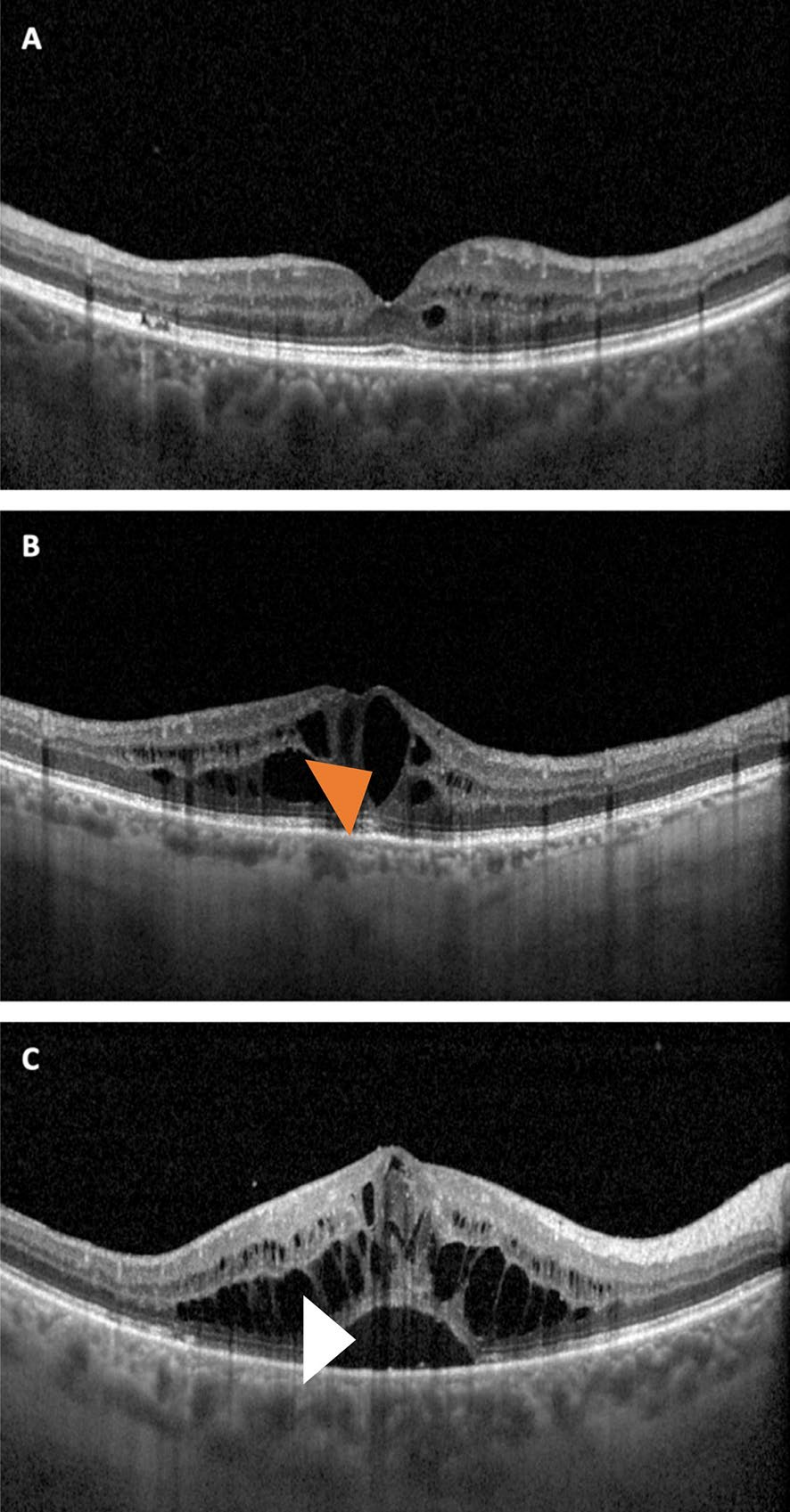
Clinical images showing structural OCT representations of the DME patterns. Representative horizontal optical coherence tomography (OCT) B-scan images through the fovea from three eyes with diabetic macular edema. OCT images were graded for qualitative features allowing characterization in different patterns that were investigated as prognostic factors influencing the longitudinal retinal thinning. Cases with intraretinal areas of reduced reflectivity were graded as being affected by diffuse macular thickening (**A**). Cases developing cystoid spaces (orange arrowhead) were graded as being characterized by cystoid macular edema (**B**). The evidence of an hyporeflective space below the neuroretina (white arrowhead) defined the presence of a subfoveal neuroretinal detachment (**C**).

Two graders also reviewed OCT images between baseline and follow-up visits in order to define the DME phenotype for each case (Fig. 1). Cases with intraretinal areas of reduced reflectivity were categorized as being affected by a diffuse macular thickening^13,14^. Conversely, cases developing cystoid spaces in the 1-mm central circle during the 3-year follow-up were graded as being characterized by cystoid macular edema^13,14^. For subfoveal neuroretinal detachment, a hyporeflective space below the neuroretina was identifiable on OCT images^13,14^.

Discrepancies between the two graders were resolved by consensus or adjudication by a third experienced grader (GQ).

### Statistical analysis

To detect departures from normality distribution, a Shapiro–Wilk’s test was performed for all variables. Means and standard deviation (SD) were computed for all quantitative variables.

The statistical significance of the differences for quantitative variables was assessed using Wilcoxon Signed Rank test. Contributory factors affecting inner and outer retina thickness changes were examined using a univariable and multivariable linear mixed model. Multivariable models were constructed including the following potential confounding factors: age, gender and any other parameters in which the p value was < 0.1 in a univariable analysis.

The Statistical Package for Social Sciences (version 23.0, SPSS Inc., Chicago, IL, USA) was used for statistical analysis. The chosen level of statistical significance was *p* < 0.05.

## Results

Overall, 56 eyes from 50 diabetic subjects (29 males, 21 females) met our inclusion criteria and were enrolled in this longitudinal study. Mean ± SD age at baseline was 61.4 ± 16.8 years. The visual acuity was 0.27 ± 0.28 LogMAR and 0.26 ± 0.25 LogMAR at baseline and study visits, respectively (*p* = 0.114). Mean ± SD number of intravitreal anti-VEGF injections before baseline visit was 4.8 ± 4.2. During the follow-up period within baseline and study follow-up visits, the mean ± SD number of intravitreal anti-VEGF injections was 8.1 ± 6.0.

Of these eyes, the DME appearance of 13 eyes were classified as being characterized by the development of SND during the 3-year follow-up (SND phenotype), while 27 cases had evidence of CME within the follow-up period (CME phenotype). Eight out of the 13 cases with the SND phenotype were also characterized by evidence of CME throughout the follow-up. Finally, neither SND nor CME was identified in 23 eyes which were categorized as being affected by a diffuse macular thickening throughout the 3-year follow-up. Considering the whole cohort of patients (regardless of the OCT pattern identified), mean ± SD number of SND occurrences during the study period (36 months) was 0.39 ± 0.89 [range 0–4], while mean ± SD number of CME occurrences during the study period was 1.53 ± 2.35 [range 0–8].

### Longitudinal changes in inner and outer retinal thickness

The perifoveal inner retinal thickness was 161.9 ± 28.9 μm at baseline and 152.6 ± 29.9 μm at the study follow-up visit (*p* < 0.0001). Similarly, the perifoveal outer retinal thickness was 94.6 ± 14.6 μm at the baseline visit and was significantly reduced at the study follow-up visit (88.5 ± 12.7 μm, *p* < 0.0001).

The parafoveal inner retinal thickness was 166.2 ± 28.8 μm and 154.5 ± 32.9 μm at the baseline and final follow-up visits, respectively (*p* < 0.0001). The parafoveal outer retinal thickness was 101.4 ± 18.9 μm at baseline and was significantly reduced at the study follow-up visit (96.3 ± 15.9 μm, *p* = 0.001).

In the foveal region, the inner retinal thickness was 91.1 ± 30.3 μm at baseline and 85.5 ± 31.3 μm at the study follow-up visit (*p* = 0.027). Uniformly, the outer retinal thickness was 100.9 ± 26.9 μm and 97.4 ± 26.0 μm at the baseline and final follow-up visits, respectively (*p* = 0.044).

The rate of perifoveal thinning was − 2.9 ± 4.9 μm/year for the inner retina and − 1.8 ± 3.1 μm/year for the outer retina. Similarly, the rate of parafoveal thinning was − 3.5 ± 5.5 μm/year for the inner retina and − 1.3 ± 3.0 μm/year for the outer retina. The rate of foveal thinning was − 1.5 ± 5.0 μm/year for the inner retina and − 0.5 ± 3.7 μm/year for the outer retina.

### Multivariable analysis on factors associated with rate of retinal thinning

Factors contributing to the rate of inner and outer retinal thinning among the subjects are shown in Table 1. In the multivariable analysis, type of diabetes (type 2 vs. type 1) was associated with increased foveal inner retinal thinning (*p* = 0.019). A higher number of SND recurrences during follow-up (*p* = 0.006) was associated with statistically significant faster foveal outer retinal thinning. Type 2 diabetes (*p* < 0.0001), higher age (*p* = 0.033) and CME phenotype (*p* = 0.040) were associated with increased parafoveal inner retinal thinning. Gender (males vs. females, *p* = 0.003) and DR stage (NPDR vs. PDR, *p* = 0.013) were associated with statistically significant faster perifoveal inner retinal thinning, while DR stage (NPDR vs. PDR, *p* = 0.036) was associated with increased perifoveal outer retinal thinning.

**Table 1:** Factors contributing to the rate of inner and outer retinal thinning by univariable and multivariable mixed model analysis.

	Inner retina				Outer retina			
	Univariable		Multivariable		Univariable		Multivariable	
	β, 95% CI	*P* value	β, 95% CI	*P* value	β, 95% CI	*P* value	β, 95% CI	*P* value
Fovea								
Age	0.187 (1.398)	.168	–	–	− 0.039 (− 0.289)	.773	–	–
Gender: M/F	0.231 (1.748)	.086	0.150 (1.050)	.299	− 0.213 (− 1.600)	.115	–	–
Type of diabetes: 2/1	0.369 (2.921)	.005	**0.111 (1.847)**	**.019**	0.101 (0.748)	.458	–	–
DR stage: PDR/NPDR	− 0.168 (− 1.252)	.216	–	–	0.262 (1.992)	.051	0.192 (1.565)	.124
Number of anti-VEGF injections	0.075 (0.555)	.581	–	–	− 0.187 (− 1.399)	.168	–	–
Number of intravitreal treatments	0.128 (0.952)	.345	–	–	− 0.232 (− 1.753)	.085	− 0.203 (− 1.409)	.165
SND phenotype	− 0.036 (− 0.263)	.794	− 0.061 (− 0.204)	.839	− 0.371 (− 2.936)	.005	0.294 (1.172)	.247
Number of SND during follow-up	− 0.060 (− 0.445)	.658	0.038 (0.128)	.899	− 0.484 (–4.063)	< .0001	**− 0.696 (− 2.852)**	**.006**
CME phenotype	0.057 (0.422)	.675	0.163 (0.890)	.378	− 0.087 (− 0.644)	.522	0.154 (0.847)	.401
Number of CME during follow-up	− 0.027 (− 0.200)	.842	− 0.109 (− 0.592)	.556	− 0.180 (− 1.347)	.184	− 0.088 (− 0.525)	.602
Parafovea								
Age	0.287 (2.205)	.032	**− 0.450 (− 2.196)**	**.033**	0.023 (0.1707)	.865	–	–
Gender: M/F	0.208 (1.562)	.124	–	–	− 0.279 (− 2.138)	.037	− 0.240 (0.124)	.081
Type of diabetes: 2/1	0.510 (4.360)	< .0001	**0.796 (4.030)**	** < .0001**	− 0.094 (− 0.692)	.492	–	–
DR stage: PDR/NPDR	− 0.233 (− 1.764)	.083	− 0.184 (− 1.293)	.202	− 0.040 (− 0.294)	.770	–	–
Number of anti-VEGF injections	− 0.003 (− 0.022)	.983	–	–	− 0.118 (− 0.873)	.387	–	–
Number of intravitreal treatments	0.103 (0.762)	.449	–	–	− 0.153 (− 1.134)	.262	–	–
SND phenotype	0.045 (0.333)	.741	0.157 (0.628)	.533	− 0.135 (− 1.000)	.322	− 0.241 (− 1.779)	.437
Number of SND during follow-up	− 0.071 (− 0.521)	.605	− 0.148 (− 0.589)	.559	− 0.236 (− 1.787)	.080	− 0.400 (− 1.440)	.156
CME phenotype	0.330 (2.960)	.021	**0.344 (2.108)**	**.040**	0.001 (0.010)	.992	0.227 (1.245)	.219
Number of CME during follow-up	− 0.004 (− 0.031)	.975	− 0.154 (− 0.943)	.350	− 0.131 (− 0.969)	.337	− 0.210 (− 1.141)	.259
Perifovea								
Age	0.253 (1.922)	.060	− 0.120 (− 0.563)	.576	0.261 (1.987)	.052	0.002 (0.015)	.998
Gender: M/F	0.414 (3.338)	.002	**0.409 (3.100)**	**.003**	0.082 (0.606)	.547	–	–
Type of diabetes: 2/1	0.307 (2.371)	.021	0.145 (0.671)	.505	0.175 (1.302)	.198	–	–
DR stage: PDR/NPDR	− 0.032 (− 2.558)	.012	**− 0.377 (− 2.567)**	**.013**	− 0.320 (− 2.480)	.016	**− 0.339 (− 2.512)**	**.036**
Number of anti-VEGF injections	0.005 (0.036)	.971	–	–	− 0.257 (− 1.953)	.056	− 0.284 (− 2.216)	.051
Number of intravitreal treatments	0.092 (0.679)	.500	–	–	− 0.182 (− 1.360)	.180	–	–
SND phenotype	0.129 (0.995)	.334	0.292 (1.073)	.289	0.017 (0.128)	.899	0.273 (0.992)	.326
Number of SND during follow-up	0.073 (0.540)	.592	− 0.297 (− 1.076)	.287	− 0.101 (− 0.748)	.458	− 0.420 (− 1.552)	.127
CME phenotype	0.204 (1.530)	.132	0.256 (1.517)	.136	0.038 (0.279)	.781	0.254 (1.339)	.187
Number of CME during follow-up	0.139 (1.033)	.306	− 0.099 (− 0.589)	.559	0.034 (0.251)	.802	− 0.012 (− 0.065)	.949

Values are shown in β coefficient (95% confidence interval), unless otherwise indicated.

*M* males, *F* females, *DR* diabetic retinopathy, *PDR* proliferative diabetic retinopathy, *NPDR* non-proliferative diabetic retinopathy, *VEGF* vascular endothelial growth factor, *SND* subfoveal neuroretinal detachment, *CME* cystoid macular edema.

Statistically significant *p* values shown in bold.

## Discussion

In this study, DME phenotypes were associated with the rates of inner and outer retinal thinning in patients undergoing anti-VEGF treatment. The number of subfoveal neuroretinal detachment recurrences during the study period was associated with a faster thinning of the outer retina. Similarly, the CME phenotype showed faster rates of parafoveal inner retinal thinning. Since the aim of this study was to assess the associations between DME phenotypes and longitudinal structural modifications of the macula, whose OCT evaluation could be altered by co-existing macular disorders or the presence of intraretinal fluid, we excluded subjects with vitreomacular disorders and those without resolution of DME after 3 years of follow-up in at least one visit.

In a previous study using structural OCT, our group demonstrated that the evidence of specific quantitative modifications of the outer retina was correlated with a worse long-term visual acuity in DME eyes undergoing anti-VEGF treatment^12^. In details, the latter study enrolled patients with an extended follow-up (i.e. > 5 years) and confirmation of DME resolution in at least one assessment (study visit) after five years of visits following the initiation of anti-VEGF intravitreal treatment. Fifty patients were analyzed and two subgroups (i.e. 24 eyes with a visual acuity lower than 20/40 constituted the “poor/intermediate vision” group, and 37 eyes with a visual acuity higher than 20/40 were included in the “good vision” group). In the latter study^12^, the neuroretina was qualitatively and quantitatively assessed with a topographic analysis in order to detect changes occurring at the level of the inner and outer retina. The latter study^12^ demonstrated that the thicknesses of the foveal and parafoveal outer retina are significantly lower in subjects with worse long-term visual outcomes.

In order to expand the above-mentioned findings^12^, in the present study we add to the literature by reporting on associations between DME phenotypes and rates of inner and outer retinal thinning in patients undergoing anti-VEGF treatment. As specified above, DME may manifest with different morphological patterns on structural OCT. One of the most notable observations from our study was that the number of SND recurrences occurring during the follow-up was associated with a greater outer retinal thinning in the foveal region. Our results are in agreement with Vujosevic et al.^23^ who demonstrated that presence of neuroretinal detachment preceding the study visit correlated with a more severe impairment of the outer retina. Our results seem to corroborate the latter aspect by providing longitudinal data. We may speculate that the accumulation of subretinal fluid may cause a mechanical damage to photoreceptors this eventually resulting in a longitudinal thinning of the outer retina. Importantly, the number of SND during the follow-up period rather than the SND pattern was associated with a greater thinning of the foveal outer retina. The latter aspect may suggest that the damage may be the result of a persevering mechanical tension to the outer retina caused by the presence of subretinal fluid.

A previous paper has investigated the longitudinal changes in the inner retinal thickness in eyes treated for DME during a 1-year follow-up^12^. The latter study demonstrated a progressive thinning of the inner retina in these eyes with a considerable impact on retinal function. We confirmed that the inner retinal layers progressively reduce in DME eyes, and this reduction does not appear to be influenced by treatment characteristics (i.e. number of intravitreal treatments). As specified above, DME may be characterized by cystoid spaces (i.e. cystoid macular edema) that appear as round- or oval-shaped areas of lower reflectivity with highly reflective septa isolating these intraretinal cavities accommodating fluid. In our study cohort, the presence of a CME pattern was associated with a greater longitudinal parafoveal inner retinal thinning. These results seem to suggest that the presence of large cavities within the retina may cause an irreversible damage to cells located in the inner retina. There are two potential hypotheses which may explain this association: (i) postreceptor functional loss, and (ii) mechanical tension. According to the postreceptor functional loss hypothesis, the neuronal damage may be a consequence of impeded axoplasmic transport secondary to the presence of cysts. Alternatively, the mechanical tension to the inner retina caused by the presence of large cysts may lead to a subsequent loss of inner retinal cells.

We also found that the type of diabetes is significantly associated with the rate of inner retinal thinning in patients undergoing anti-VEGF treatment. In details, patients with type 2 diabetes were characterized by a faster inner retinal thinning, as compared with type 1 diabetic subjects. Our results are in agreement with previous evidences suggesting that type 2 diabetes may be characterized by a greater thinning of the inner retina^24^. Furthermore, patients with a diagnosis of PDR were characterized by a faster thinning of the inner and outer retina in the perifoveal region. The latter finding may be secondary to the panretinal photocoagulation treatment that was performed in these patients.

Our study has limitations that must be taken into account. First, our retrospective investigation could be prone to selection and ascertainment bias. Furthermore, the included patients were not followed up at regular intervals as our cohort was not part of a large trial. However, we only included subjects who had at least three yearly retinal visits over a period of three years after the 1^st^ evidence of DME resolution and this may have restrained this limit. Second, DME classification was based on subjective grading. To address this limitation, two graders were tasked with classifying DME phenotypes and the final grading was obtained after open adjudication. Third, other uncontrolled differences in ocular and systemic features among DME phenotype groups might have affected the retinal measurements.

In conclusion, the present study investigated the association between DME patterns and long-term rate of inner and outer retinal thinning in DME eyes treated with anti-VEGF therapy. Our results appear to suggest that clinical characteristics, including DME phenotypes, may affect both the inner and outer retinal thinning. While presence of large cystoid spaces may determine an inner retinal thinning, the development of SND seems to be associated with a longitudinal thinning of the outer retina. These findings may help broaden our knowledge regarding the natural history of DME and the evolution of retinal damage in these eyes.

## Data Availability

The data used to support the findings of this study are available from the corresponding author upon request.
